# Association of Maternal Obesity and Neonatal Hypoxic-Ischemic Encephalopathy

**DOI:** 10.3389/fped.2022.850654

**Published:** 2022-04-29

**Authors:** Meredith Monaco-Brown, Upender Munshi, Michael Joseph Horgan, Jamie L. Gifford, Rubia Khalak

**Affiliations:** Department of Pediatrics, Bernard and Millie Duker Children’s Hospital at Albany Medical Center, Albany, NY, United States

**Keywords:** maternal obesity, neonatal hypoxia, HIE, perinatal encephalopathy, HIE (hypoxic ischemic encephalopathy), BMI—body mass index

## Abstract

**Objective:**

More women are obese at their first prenatal visit and then subsequently gain further weight throughout pregnancy than ever before. The impact on the infant’s development of neonatal hypoxic ischemic encephalopathy (HIE) has not been well studied. Using defined physiologic and neurologic criteria, our primary aim was to determine if maternal obesity conferred an additional risk of HIE.

**Study Design:**

Data from the New York State Perinatal Data System of all singleton, term births in the Northeastern New York region were reviewed using the NIH obesity definition (Body Mass Index (BMI) ≥ 30 kg/m2). Neurologic and physiologic parameters were used to make the diagnosis of HIE. Physiologic criteria included the presence of an acute perinatal event, 10-min Apgar score ≤ 5, and metabolic acidosis. Neurologic factors included hypotonia, abnormal reflexes, absent or weak suck, hyperalert, or irritable state or evidence of clinical seizures. Therapeutic hypothermia was initiated if the infant met HIE criteria when assessed by the medical team. Logistic regression analysis was used to assess the effect of maternal body mass index on the diagnosis of HIE.

**Results:**

In this large retrospective cohort study we evaluated outcomes of 97,488 pregnancies. Infants born to obese mothers were more likely to require ventilatory assistance and have a lower 5-min Apgar score. After adjusting for type of delivery and maternal risk factors, infants of obese mothers were diagnosed with HIE more frequently than infants of non-obese mothers, OR 1.96 (1.33–2.89) (*p* = 0.001).

**Conclusion:**

Infants of obese mothers were significantly more likely to have the diagnosis of HIE.

## Introduction

Obesity remains an escalating problem for all ages, genders or socioeconomic backgrounds in the United States and worldwide. An increase over the past three decades has also been noted in the pregnant population. More women are obese at their first prenatal visit and then subsequently continue to gain more weight during pregnancy (1–3). Although obesity rates are rising in all women of childbearing age, it does not affect all races or socioeconomic groups similarly. Obesity rates vary widely by race, with significantly higher rates in Black and Hispanic women vs. White or Asian Non-Hispanic women. Obesity rates are also higher for people with lower education levels and in less urbanized regions (4). Maternal obesity can lead to health problems for the mother, the pregnancy and labor process, and subsequent neonatal complications (3, 5–7). Studies have shown that high-risk pregnant women who are also obese can have further worsening of their underlying disease, increasing neonatal risk and the need for admission to the neonatal intensive care unit (NICU) (8–12).

Global prevalence of obesity is also rising (13–15), and significant research internationally has illuminated the effects of maternal obesity. A comprehensive review from the United Kingdom showed that more than half of the pregnancy-related deaths in all mothers occurred in obese women (6). Labor complications included an increase in assisted, instrumental delivery and a significantly higher rate of cesarean delivery. The incidence of cesarean section remained higher even when adjusted for potential confounders such as preeclampsia, diabetes and macrosomia. The most common neonatal problems associated with maternal obesity include macrosomia with a birthweight greater than four kilograms and hypoglycemia. Recently Rimsza et al. published a study demonstrating lower umbilical arterial pH levels in obese women undergoing scheduled C-section, which was attributed to the influence of obesity on both spinal anesthesia placement and related hypotension, as well as slower time to delivery (16). This is highly concerning considering the increased rates of C-section in obese mothers. Other newborn complications include congenital anomalies such as neural tube defect but whether this is diet or folic acid related has not been determined (10, 11).

Cnattingius et al. as well as several other researchers have shown that obesity places the mother-infant dyad at an increased risk during pregnancy, delivery and postpartum (11, 16–20). The inability to reliably perform fetal monitoring and assess fetal wellbeing at an early stage compounds the increased risk to infants of obese mothers. In 2017, Villamor et al. evaluated the impact of maternal body mass index (BMI) and diabetes status on the risk of asphyxia-related complications to the neonate (21). They found that low Apgar scores as a surrogate for the diagnosis of hypoxic ischemic encephalopathy (HIE) were increased in infants whose mothers had diabetes and were obese.

In recent years, there has been literature directly associating HIE with maternal overweight or obese status (22). One study from a single large academic institution describes more than a doubled rate of HIE in babies born to obese women after adjusting for race, from a total number of 27 babies treated for HIE (23). Our group has briefly commented on our efforts to gather regional data associating obesity with HIE (24). The literature describes several of the ramifications of maternal obesity on the neonate, but whether maternal obesity confers an additional risk to the infant on the development of HIE using both physiologic and neurologic criteria has not been described on a large scale.

In this population-based cohort study we evaluated whether neonates are at higher risk of HIE when born to mothers who are obese. Our primary aim was to determine if maternal obesity is associated with the diagnosis of HIE when controlling for the confounding factors of maternal diabetes, hypertension and mode of delivery.

## Methods

This retrospective, population-based cohort study was approved by the Institutional Review Board of Albany Medical College (AMC). Our regional level 4 NICU serves as the HIE referral/treatment center covering a large area of Northeast New York State serving approximately 2.7 million people. On average we have about 14,000 births in our region per year. The surrounding hospitals with the delivery of an infant with concern for possible HIE are encouraged to contact us for consultation and consideration for therapeutic hypothermia as treatment for HIE.

We reviewed the de-identified maternal and neonatal information from the New York State Perinatal Data System, a database that collects mandated reporting data on mothers and newborns from all New York State hospitals. The maternal/neonatal data included for this study was for all live born singleton births of 36 0/7–42 0/7 weeks gestation delivering in the Northeastern New York (NENY) 18 hospitals in the 17 counties of upstate New York from January 1, 2011 to December 31, 2017. Perinatal transfer and affiliation agreements exist between the regional perinatal center, AMC and the other hospitals in the NENY region. The agreements incorporate data collection and evaluation of each hospital by utilizing the statewide perinatal data system. For the purposes of this study, National Institute of Health BMI classification was used with obesity defined as a BMI ≥ 30 kg/m^2^. When obesity levels were further stratified, the NIH classification was used with obese level I, BMI 30–34.9 kg/m^2^, obese level II or severely obese, BMI 35–39.9 kg/m^2^, and obese level III or morbidly obese BMI ≥ 40 kg/m^2^ (25).

Data were collected based on availability and relevancy to both maternal obesity and risk of HIE. Variables collected included maternal characteristics such as parity, pre-pregnancy BMI, co-morbidities of pre-pregnancy diabetes mellitus (DM), gestational diabetes, pre-pregnancy hypertension, gestational hypertension, chorioamnionitis, and demographics of maternal age, race and marital status. Delivery characteristics included prolonged premature rupture of membranes, mode of delivery including operative vaginal delivery and cesarean section. Infant data collection recorded gestational age (GA), birth weight (BW), gender, Apgar scores, delivery room (DR) resuscitation defined as the need for positive pressure with nasal continuous positive airway pressure (NCPAP) or endotracheal ventilation and the need for respiratory support beyond 6 h of age defined as the need for positive pressure with NCPAP or higher mode of ventilation.

Diagnoses of possible HIE and definitive HIE were recorded. Possible HIE was defined as a 5-min Apgar score ≤ 5, having received assisted ventilation after delivery and gestational age ≥ 36 weeks. The diagnosis of definitive HIE was made by evaluation of both physiologic and neurologic criteria. Physiologic criteria included the presence of an acute perinatal event such as a significant fetal bradycardia, cord prolapse, placental abruption or a uterine rupture, a 10-min Apgar score ≤ 5, and the finding of acidosis either fetal with cord pH ≤ 7.1 or infant with blood gas pH ≤ 7.1 by 60 min of age or base deficit of ≥ 12. The neurologic factors on evaluation for HIE included hypotonia, abnormal reflexes, absent or weak suck, hyperalert or irritable state or evidence of clinical seizures. Therapeutic hypothermia was initiated if the infant met HIE criteria, was ≥ 36 weeks gestation, ≤ 6 h of age and assessed by the NICU medical team to have encephalopathy, defined as having the abnormal neurological exam findings noted above.

### Statistical Analysis

**FIGURE 1:**
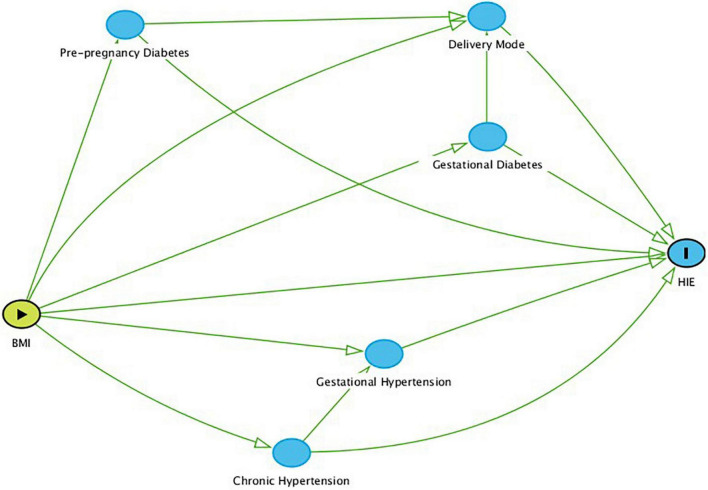
Direct Acyclic Graph (DAG) based on known relationships between BMI, HIE, and the confounders addressed in this report. Nodes represent variables (including confounders), and arrows reflect causal relationships.

Demographic characteristics as well as maternal and neonatal diagnoses were compared for mothers and infants after division into two BMI groups, non-obese BMI < 30 or obese BMI ≥ 30. Statistical analysis of normatively distributed continuous data was done using the Student *t*-test and Chi-squared test was used for analyses of categorical data. Logistic regression was used to evaluate the effect of maternal BMI on the diagnosis of HIE while controlling for potential confounders such as pre-pregnancy diabetes mellitus, gestational diabetes, pre-pregnancy hypertension, gestational hypertension, and mode of delivery. The relationship between BMI, HIE, and these confounders is described in Figure 1. The level of significance was established at the *p* < 0.05 level. All statistical analyses was done using Stata Software, version 12.2, StataCorp. 2017. *Stata Statistical Software: Release 15*. College Station, TX: StataCorp LLC (Stata, RRID:*SCR_012763*).

## Results

**FIGURE 2:**
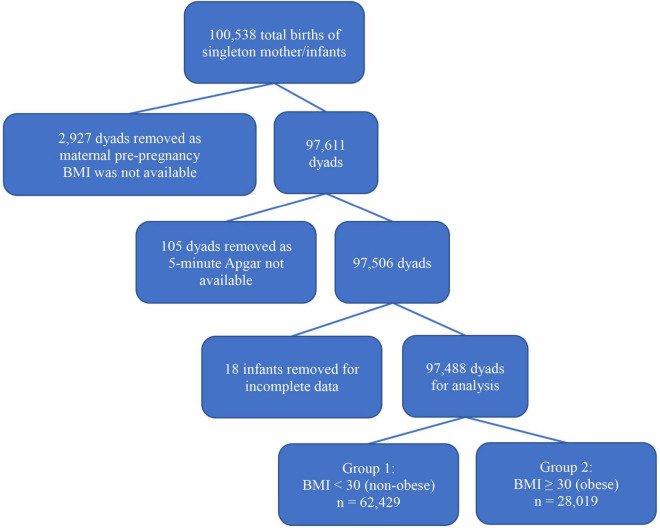
Flow diagram of mother-infant selection.

Our initial data capture for all live born singleton births between 36 0/7 weeks and 42 0/7 weeks gestation resulted in 100,538 mother-infant dyads. Pre-pregnancy BMI was not available for 2,927 women and these dyads were removed from further review. Of the resultant 97,611 mother-infant dyads, 123 dyads were removed for incomplete infant record data leaving 97,488 mother-infant dyads for analysis. The mother-infant pairs were separated into two groups, Group 1, the non-obese group with maternal BMI < 30 and Group 2, the obese group with maternal BMI ≥ 30. Figure 2 shows the flow diagram of the attrition during data collection.

Upon analysis of over 97,000 dyads, pre-gestational and gestational DM along with pre-pregnancy hypertension and gestational hypertension were significantly more likely to be noted in the obese women (*p* < 0.001). Significantly more women in the obese group delivered by cesarean section when compared to non-obese women, (*p* < 0.001). Infants born to obese mothers had a higher BW, 3,502 grams vs. 3,387 g (*p* < 0.001), and more infants required DR resuscitation after delivery. The number of infants receiving a 5-min Apgar of ≤ 5 was significantly higher in those born to obese mothers. Detailed demographic data is available in a prior publication from this group (24). Our data shows a steady notable increase in the incidence of maternal obesity throughout the years of the study (Table 1).

**TABLE 1:** Hypoxic ischemic encephalopathy and hypothermia use by year.

Year	Total births ≥ 36 weeks	Births ≥ 36 weeks non- obese mothers	Births ≥ 36 weeks obese mothers (% total)	Possible HIE	Possible HIE non-obese mothers	Possible HIE obese mothers	Diagnosis of HIE	Use of therapeutic hypothermia
2011	14,991	10,872	4,119 (27.4)	45	29	16	18	8
2012	14,675	10,667	4,008 (27.5)	40	23	17	5	3
2013	14,531	10,504	4,027 (27.8)	70	49	21	16	9
2014	14,140	10,120	4,020 (28.4)	63	44	19	22	18
2015	14,187	9,973	4,214 (29.7)	64	40	24	20	14
2016	14,003	9,732	4,271 (30.7)	70	44	26	24	20
2017	13,720	9,399	4,321 (31.5)	81	49	32	47	45

Analysis of our data also revealed that obese mothers are significantly more likely to have infants in both the possible HIE and diagnosed HIE groups (*p* = 0.001) (24). When logistic regression analysis was performed controlling for maternal pre-gestational DM, gestational DM, pre-gestational hypertension, gestational hypertension, and mode of delivery, the diagnosis of HIE was seen more frequently in infants of obese mothers than infants of non-obese mothers, raw OR = 1.88 (1.25–2.82), adjusted OR = 1.73 (1.13–2.65) (*p* = 0.001). Maternal BMI was stratified by the NIH BMI categories to assess the impact of BMI by subgroup on the incidence of HIE. When the subcategories of obese BMI, levels 1, 2, and 3 were compared and no difference in the incidence of HIE was noted (Table 2). Obese BMI is associated with an HIE diagnosis, but further increases in BMI did not appear to exert an additive effect. When we reviewed the characteristics of infants who received an HIE diagnosis, we found that 53% (56 of the 106 infants) required endotracheal tube placement in the delivery room, 27% required chest compressions and 15% of the infants received epinephrine during their delivery room resuscitation. Fetal acidosis was noted on cord blood gas with cord pH 6.91 ± 0.19 (mean, *SD*).

**TABLE 2:** Infants with HIE by Maternal NIH-BMI category.

BMI category	Number of infants	HIE	Pre-pregnancy diabetes	Gestational diabetes	Chronic hypertension	Gestational hypertension	Cesarean delivery
Underweight	2,786	3 (0.1)	2 (0.1)	83 (3.0)	17 (0.6)	64 (2.3)	531 (19.1)
Normal	40,848	37 (0.1)	134 (0.3)	1,553 (3.8)	298 (0.7)	1,186 (2.9)	10,123 (24.8)
Overweight	25,795	21 (0.1)	160 (0.6)	1,585 (6.1)	408 (1.6)	1,232 (4.7)	7,948 (30.8)
Obese (All classes)	28,019	46 (0.2)	424 (1.5)	3,129 (10.8)	1,136 (3.9)	2,369 (8.2)	11,701 (40.5)
Class I	14,588	22 (0.2)	144 (1.0)	1,346 (9.2)	400 (2.7)	958 (6.6)	5,479 (37.6)
Class II	7,714	12 (0.2)	129 (1.7)	893 (11.6)	309 (4.0)	728 (9.4)	3,314 (43.0)
Class III	5,717	12 (0.20)	151 (2.6)	427 (15.6)	427 (7.5)	683 (12.0)	2,908 (50.9)

## Discussion

This population-based retrospective cohort study demonstrates that infants of obese mothers were significantly more likely to have the diagnosis of HIE. What is known concerning the association of maternal obesity and various neonatal outcomes remains limited. Investigators have found that the infants born to obese mothers had higher birth weights and were more likely to be admitted to the NICU when compared to non-obese women (26). Reasons for NICU admission were not given, although possibilities for admission include hypoglycemia secondary to macrosomia, temperature instability prompting a sepsis evaluation or respiratory distress from retained lung fluid. Another reason that has been previously reported is premature birth, which occurs more frequently with obese mothers (11). Like other researchers, we found that infants born to obese women were heavier than infants born to non-obese women. We also discovered that infants of obese mothers were much more likely to require assisted respiratory ventilation after delivery (18).

Persson et al. showed an increasing incidence of HIE among infants born to obese or overweight mothers using 5 and 10-min Apgar scores of 0–3 as a surrogate for the diagnosis of HIE (19). The group noted these results not only in crude odds ratios but also after adjustment for certain socioeconomic factors and comorbidities of diabetes and hypertension. A recent study by Smid et al. revealed that when comparing urgent and elective cesarean section, more urgent operative deliveries occurred in normal BMI women, as many obese women had scheduled cesarean delivery. The researchers used Apgar score < 5 as a surrogate for HIE when assessing neonatal morbidity in infants born to obese mothers (27). This is pertinent to Rimsza et al.’s findings of increased anesthetic and surgical issues leading to decreased cord pH in women undergoing scheduled C-section, as previously mentioned (16). Chen and colleagues found no correlation with maternal obesity and Apgar score of <3 (28). This establishes an advantage of our study, as the Apgar score alone may not be indicative of HIE nor can it be used exclusively as a surrogate for HIE. Investigators have reported that an increase in the risk of fetal acidosis was noted in overweight and obese women who are at a higher risk for delivery complications (29). The specific pathophysiologic mechanisms of why infants of obese women are more susceptible to HIE have not been fully elucidated. Teo et al. have shown with a neonatal brain injury rat model that maternal obesity resulted in an exacerbation of inflammatory damage seen in neonatal HIE (30). Another possible etiology could be vascular changes in the placenta leading to vasoactive responses and periods of limited blood flow to the fetus as can be seen in pregnant women with hypertension either chronically or during gestation (31). The hypotension associated with spinal anesthesia which is more common in obese women may also adversely affect perfusion to the fetus (16). Another possibility is the effect of excessive glucose movement across the placenta in obese women as is seen in women with pre-gestational DM and gestational DM (32, 33). This excess glucose load effects both the placenta and the fetus leading to large infant size and increased risk of delivery and post-delivery concerns. We adjusted for the confounders of diabetes and hypertension in regression analysis thereby showing that maternal obesity is an independent risk factor or the development of HIE.

There has been an increase in the possible HIE infants in our region over the study years which has been coupled with an increase in infants receiving an HIE diagnosis and then subsequently an increase in the number of infants treated with therapeutic hypothermia (Table 1). This increase was more evident in years 2014–2017 of the study. The most prominent increase was noted in 2017 when a concerted education effort had been made during perinatal outreach to assist in identifying HIE or possible HIE infants earlier. We had also begun to cool infants receiving the diagnosis of milder HIE, in part based on recent evidence of adverse neurologic outcomes in infants diagnosed with mild HIE (34, 35). There was no significant difference in the number of total singleton births ≥ 36 weeks from year to year of the study, yet there was a steady rise in the number of infants born to obese mothers, also particularly in the last 4 years of the study.

Baeten et al. showed that obesity is a strong risk factor for resultant pregnancy complications for not only obese women but also overweight women with BMI- 25–29.9 kg/m^2^ (5). An increase in gestational diabetes, preeclampsia, eclampsia, cesarean delivery, and macrosomia was also noted. Although we divided our groups into obese and non-obese, thereby placing overweight BMI into the non-obese group, placing overweight BMI women instead in the obese group would not have a significantly different effect. When we assessed the incidence of HIE by maternal BMI category dividing dyads into all 6 NIH BMI categories as was done in Table 2, we found that there was a very significant difference between normal BMI and obese BMI groups but not with the sub-categories of obese BMI. We also found that the incidence of HIE in the overweight BMI women was similar to the incidence of HIE in the normal BMI women.

A limitation of our study was its retrospective nature of a confining the availability of certain physiologic criteria such as cord pH and infant blood gas values. We had some missing cord pH values for infants in the HIE group and only scattered existing values for the non-HIE infants often because they were not obtained. We were easily able to identify those infants who were given the diagnosis of HIE based on physiologic and neurologic criteria, but we did not always have clear documentation in the medical record of which particular parameters qualified to make the HIE diagnosis. Another limitation was our inability to link our data to meaningful socioeconomic information, as we know there is an association of lower socioeconomic status and an increased incidence of obesity (4) and thereby possibly an increase in HIE diagnosis.

In our study, we found that infants of obese mothers are significantly more likely to receive the diagnosis of HIE. To date the literature has few studies evaluating for the association of maternal obesity and HIE. Several of those studies use either Apgar score or evidence of fetal acidosis as the sole markers for HIE. Our definition of HIE included the Apgar score as just one component, utilizing both physiologic and neurologic criteria to assign the diagnosis of definitive HIE. Even our definition of possible HIE utilized criteria beyond Apgar score, including GA and need for resuscitation. Another strength of our study is that our perinatal regional center covers an expansive geographic region of not only northeastern New York State but also western Vermont and western Massachusetts thereby assessing a large number of births. Future studies are needed to be done prospectively to ensure complete capture of physiologic laboratory data and detailed neurologic examination parameters. Understanding the incidence of maternal obesity in various communities and learning how obesity increases the risk of HIE is a crucial step toward implementing targeted prevention prenatally and perinatally for all affected pregnancies.

## Data Availability Statement

The data analyzed in this study is subject to the following licenses/restrictions: Data is only available by approved request to the NYS Department of Health and the NYS Department of Vital Records. Requests to access these datasets should be directed to JG.

## Ethics Statement

The studies involving human participants were reviewed and approved by the Albany Medical College Institutional Review Board. Written informed consent for participation was not required for this study in accordance with the national legislation and the institutional requirements.

## Author Contributions

RK, MH, and UM contributed to conception and design of the study. JG organized the database. JG and RK performed the statistical analysis. RK wrote the first draft of the manuscript. MM-B and RK wrote sections of the manuscript. All authors contributed to manuscript revision, read, and approved the submitted version.

## Conflict of Interest

The authors declare that the research was conducted in the absence of any commercial or financial relationships that could be construed as a potential conflict of interest.

## Publisher’s Note

All claims expressed in this article are solely those of the authors and do not necessarily represent those of their affiliated organizations, or those of the publisher, the editors and the reviewers. Any product that may be evaluated in this article, or claim that may be made by its manufacturer, is not guaranteed or endorsed by the publisher.
